# Invasive lobular carcinoma of the breast: long-term prognostic value of Ki67 and histological grade, alone and in combination with estrogen receptor

**DOI:** 10.1186/2193-1801-3-70

**Published:** 2014-02-06

**Authors:** Ulrik Narbe, Pär-Ola Bendahl, Dorthe Grabau, Lisa Rydén, Christian Ingvar, Mårten Fernö

**Affiliations:** Department of Clinical Sciences, Division of Oncology, Skåne University Hospital, Lund University, SE-221 85 Lund, Sweden; Division of Surgery, Clinical Sciences, SE-221 85 Lund, Sweden; Division of Pathology, SE-221 85 Lund, Sweden; Department of Oncology, Växjö Central Hospital, SE-351 85 Växjö, Sweden

**Keywords:** Breast cancer, Invasive lobular carcinoma, Ki67, Histological grade, Prognostic factors

## Abstract

**Background:**

The aim of the present study was to investigate the long-term impact of prognostic factors in invasive lobular carcinoma (ILC) of the breast, with a primary focus on Ki67 and histological grade, alone and in combination with estrogen receptor (ER).

**Material and methods:**

One hundred and ninety two well-characterised patients with ILC were included in the study. Ki67, histological grade and ER were evaluated and combined into a prognostic index (KiGE). All grade 1 tumours and ER-positive (ER+) grade 2 tumours with Ki67 ≤ 30% were classified as low-KiGE and all the others as high-KiGE.

**Results:**

Overall, 31% of the patients have died from breast cancer. The median follow-up of the patients still alive was 21 years. Age, tumour size, axillary lymph node status (nodal status), histological grade, Ki67 and KiGE were significant prognostic factors for breast cancer mortality (BCM) in univariable analysis. In a multivariable model, adjusted for adjuvant treatment, age and progesterone receptor (PgR), the strongest prognostic factors for BCM were: Nodal status (hazard ratio (HR) = 2.9, 95% confidence interval (95% CI): 1.4-6.1), KiGE (HR = 2.0, 95% CI: 1.1-3.6), and tumour size (HR = 1.9, 95% CI: 0.98-3.8). By combining these three factors, 37% of the ILC’s could be further divided into a low-risk group, consisting of node negative small (≤ 20 mm) low-KiGE tumours, with a BCM of 5% (95% CI: 1-13%) at 10 years and 12% (95% CI: 5-22%) at 20 years follow-up. None of these patients recieved chemotherapy and only 2 recieved endocrine treatment with tamoxifen.

**Conclusions:**

The combination of Ki67, histological grade and ER into KiGE, together with tumour size and nodal status make it possible to identify a large group of ILC patients with such a good long-term prognosis that chemotherapy can be safely avoided and exclusion of endocrine therapy considered.

## Introduction

Invasive lobular carcinoma (ILC) is the second most common type of breast cancer (BC) after invasive ductal carcinoma (IDC) and comprises 5-15% of all breast cancers (Lakhani et al. 2012). The incidence of ILC appears to be increasing, particularly in postmenopausal women, and this finding may at least partly be related to hormone replacement treatment (Li et al. 2003; Reeves et al. 2006; Biglia et al. 2007). The lobular tumour cells are typically small, round, with a relatively harmless appearance, a scant cytoplasm and lack the cell adhesion molecule E-cadherin (Arpino et al. 2004; Acs et al. 2001; Rakha et al. 2010). There is a characteristic growth pattern with single-file infiltration of the benign breast tissue often without destroying normal anatomical structures (Martinez & Azzopardi 1979). Early detection by clinical examination and mammography can be difficult as ILC sometimes presents as poorly defined thickening of the breast tissue rather than as a distinct mass (Arpino et al. 2004; Singletary et al. 2005).

ILC is, compared to IDC, more often associated with higher age at diagnosis, larger tumour size, multicentricity, multifocality, bilaterality, histological grade 2, hormone receptor positivity, HER2 negativity, different metastatic pattern, lower cell proliferation rate and less responsiveness to chemotherapy (Arpino et al. 2004; Pestalozzi et al. 2008; Rakha et al. 2008a; Wiesner et al. 2009; Petrelli & Barni 2013). In spite of this, treatment strategies for ILC are often similar to those of other breast cancers, where most are of ductal origin. Three large recent studies, with long follow-up, comparing ILC and IDC show equal, or a trend towards slightly better, short-term outcome for ILC, followed by a higher incidence of late recurrences and a tendency to worse long-term outcome for ILC (Arpino et al. 2004; Pestalozzi et al. 2008; Rakha et al. 2008a). Age, tumour size, axillary lymph node status (nodal status), estrogen receptor (ER), progesterone receptor (PgR), histological grade and HER2, are widely accepted factors for the assessment of the prognosis and adjuvant treatment decision-making in breast cancer. In addition, the proliferation marker Ki67 was introduced as an important prognostic factor in the 2009 St Gallen guidelines (Goldhirsch et al. 2009). Studies presenting data on ILC show that the most powerful prognostic factors for this histological subtype are nodal status and tumour size (Arpino et al. 2004; Wasif et al. 2010; Sastre-Garau et al. 1996; Frost et al. 1995; Orvieto et al. 2008; Rakha et al. 2008b; Moreno-Elola et al. 1999). Histological grade has an important independent value in predicting outcome in IDC, but its prognostic impact in ILC is less clear (Rakha et al. 2008b; Wachtel et al. 2010; Talman et al. 2007; Sinha et al. 2000). The long-term prognostic value of Ki67 in ILC has not yet been fully studied. Recent studies by our group have shown the strong prognostic impact of an index (KiGE) combining Ki67, histological grade and ER in primary breast cancer, including all histological subtypes (Klintman et al. 2010; Strand et al. 2011,2013). The aim of the present study was to investigate the long-term prognostic value of Ki67 and histological grade alone and in combination with ER (KiGE), in a consecutive series of breast cancer patients with ILC and available data on long-term follow-up.

## Material and methods

### Patients

Between 1980 and 1991 a total of 264 cases of female breast cancer were coded as ILC at the Department of Pathology, Lund University Hospital, Sweden. Most patients were diagnosed before the start of the public mammography screening which was initiated in 1989 (Olsson et al. 2000).

**Figure 1:**
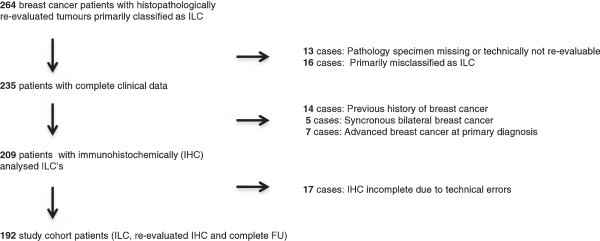
**Consort diagram.**

Histopathological re-evaluation was made of all tumours by two pathologists (II, PB), without any knowledge of patient history. Thirteen patients with missing or technically not re-evaluable pathology specimens and 16 with tumours initially misclassified as ILC’s, were excluded. Clinical data revealed 7 patients with metastatic disease at the time of diagnosis, 14 with a previous history of breast cancer, and 5 with synchronous bilateral breast cancer. These patients were also excluded. Patients who subsequently developed a new breast cancer (independent of histopathological subtype) were included. Immunohistochemical analyses were carried out on the formalin-fixed paraffin embedded tumour samples. Due to technical errors another 17 patients were excluded. Finally 192 patients, with histopathologically re-evaluated ILC, complete clinical data, and results from ER and Ki67 with immunohistochemical analyses available, were included in the study. (Consort diagram, Figure 1). Patient and tumour characteristics (Table 1) were retrieved from clinical records and pathology reports, as were follow-up data (Table 2) until November 2009. The median age was 62 years and 26% were premenopausal at the time of ILC diagnosis. Breast-conserving surgery (BCS) was performed in 40 patients (21%) and the rest had mastectomy. Axillary lymph node dissection (ALND) was performed in 181 patients (94%). The main reason for not undergoing ALND was a clinical node negative (cN-) status in patients with co-morbidity and high age at diagnosis. All patients included underwent surgery before the introduction of the sentinel node biopsy (SNB) technique. Adjuvant treatments were given in accordance with established Swedish guidelines at the time of ILC diagnosis: 93 patients (48%) recieved radiotherapy, 78 (41%) endocrine therapy and 5 (3%) chemotherapy. The histological grade was re-evaluated according to Elston and Ellis (1991) by one of the authors (DG) (Table 3).

**Table 1:** **Patient and tumour characteristics (** ***n*** **=192)**

Variables	No (%)
**Age** (years)	
Median (range)	62 (36–87)
**Menopausal status**	
Premenopausal	48 (26)
Postmenopausal	138 (74)
Unknown	6
**Type of surgery**	
BCS^a^ (no ALND^b^)	4 (2)
BCS + ALND	36 (19)
Mastectomy (no ALND)	7 (4)
Mastectomy + ALND	145 (76)
**Tumour size** (mm)	
pT1 (≤20)	108 (57)
pT2 (>20 and ≤50)	69 (37)
pT3 (>50)	12 (6)
Undefined	3
**Nodal status** ^c^	
pN- (ALND)	106 (59)
cN- (no ALND)	11
pN+	75 (41)
pN1 (1-3+)	36 (20)
pN2 (4-9+)	28 (16)
pN3 (>9+)	11 (6)
**Pathological stage** ^**d**^	
I	78 (42)
II	68 (36)
III	42 (22)
IV	0 (0)
Undefined (but not stage IV)	4
**Adjuvant therapy** ^**e**^	
RT (total)	93 (48)
CT (total)	5 (3)
ET (total)	78 (41)
RT (monotherapy)	39 (20)
CT (monotherapy)	3 (12)
ET (monotherapy)	27 (14)
RT+CT	3 (2)
RT+ET	51 (27)
CT+ET	0 (0)
RT+CT+ET	0 (0)

^a^BCS: Breast conserving surgery.

^b^ALND: Axillary lymph node dissection.

^c^c = clinical, p = pathological, N- = node negative, N+ = node positive.

^d^Pathological stage: I = pT1-2N0M0, II = T2-3N0, M0 T1-2N1M0,

III = pT3N1M0, pT1-3N2M0, T4N0-2M0, T1-4N3M0,

IV = T1-4N0-3M1.

^e^Adjuvant therapy: RT = radiotherapy, CT = chemotherapy, ET = endocrine therapy.

**Table 2:** **Recurrences and mortality**

Variables	No (%)
**New primary breast cancer**	
Contralateral	15 (8)
Ipsilateral	0 (0)
**First recurrence**	
Local	16 (8)
Regional	6 (3)
Distant	54 (28)
**Total recurrence**	76 (40)
Local	21 (11)
Regional	12 (6)
Distant	67 (35)
No recurrence	116 (61
**Cause of death**	
Breast cancer	60 (31)
Other causes	72 (38)
Alive	60 (31)

**Table 3:** **Immunohistochemical analyses and histological grade**

Variables	No (%)
**ER**	
Positive	169 (88)
Negative	23 (12)
**PgR**	
Positive	97 (51)
Negative	95 (50)
**ER/PgR**	
ER and/or PgR positive	177 (92)
ER and PgR negative	15 (8)
**HER2**	
0	153 (80)
1+	28 (15)
2+	7 (4)
3+	4 (2)
**Ki67**	
Low (0-10%)	115 (60)
Intermediate (11-30%)	62 (32)
High (>30%)	15 (8)
**Histological grade**	
1	28 (16)
2	133 (76)
3	14 (8)
Unknown	17
**Tubule formation**	
1	0 (0)
2	13 (7)
3	162 (93)
**Pleomorphism**	
1	20 (11)
2	112 (64)
3	43 (25)
**Mitotic score**	
1	155 (89)
2	10 (6)
3	10 (6)

### Immunohistochemical assay of ER, PgR, HER2 and Ki67

For all tumours, serial sections (4 μm) were cut from formalin-fixed, paraffin-embedded blocks of ILC. The blocks were sectioned and the slides were stained at one department. The sections were dried at 60°C for 1 h. After dewaxing and rehydration, the sections were treated with 10 mM citrate buffer (pH 6.0, 15 min) in a microwave oven (Shi et al. 1991). The slides were stained in an automatic immunostainer TechMate 500 (Dako) with Dako ChemMate Detection Kit peroxidase/DAB. The evaluation of the staining was performed by a pathologist (II). ER (1D5, Dako, 1:100) and PgR (Polyclonal, Dako, 1:50) positivity was defined as more than 10% stained nuclei. Ki67 (Mib-1, Immunotech, 1:100) was categorized into three groups depending on the percentage of stained nuclei: low (0-10%), intermediate (11-30%) and high (>30%). HER2 (CB11, Novocastra, 1:200) was categorized into four different immunohistochemical (IHC) groups depending on the cell membrane staining intensity: 0, 1+, 2+, 3+. A value of IHC 3+ was considered as HER2 positive (Barnes et al. 1988). A HER2 gene amplification test was not performed. All cut-offs were decided according to a predefined protocol before linking expression to survival data.

### KiGE

**Figure 2:**
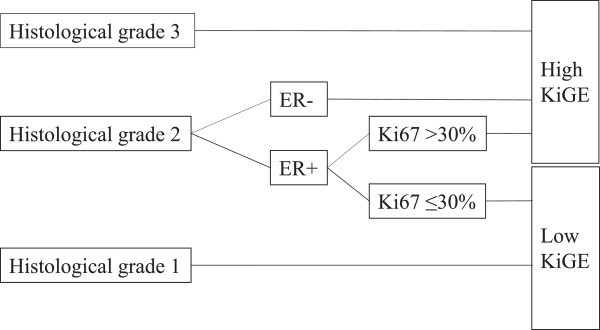
**KiGE.**

By combining Ki67, histological grade and ER, into a prognostic index (KiGE) (Strand et al. 2013), the tumours could be categorized into a KiGE low-risk (low-KiGE) and a KiGE high-risk group (high-KiGE). KiGE is based primarily on histological grade; Tumours with grade 1 are defined as low-KiGE and grade 3 as high-KiGE. The intermediate group, tumours with grade 2, is divided according to ER and Ki67 status. The ER positive (ER+) grade 2 tumours with Ki67 ≤30% are defined as low-KiGE whereas all other grade 2 tumours are defined as high-KiGE (Figure 2).

### Statistical analysis

Dependencies between Ki67, histological grade and other prognostic factors were assessed using Pearson correlation (*r*). In these analyses, the ordinal variables Ki67 and histological grade were linearly coded 1/2/3 whereas the continuous values were used for age and tumour size. All the other variables were dichotomized. The primary study endpoint was cumulative breast cancer mortality (BCM). For each patient, the follow-up time was counted from the date of surgery until death with or without breast cancer or, for the survivors, until November 2009. The log-rank test was used to compare breast cancer mortality in different strata, the trend alternative for variables with three ordered categories, and the Cox proportional hazards model for estimation of hazard ratios. Proportional hazard assumptions were checked graphically. All tests were two-sided and p-values <0.05 were considered significant. In the survival analyses, histological grade, Ki67, and the number of positive lymph nodes were analyzed as factor variables on three levels (2 degrees of freedom) with the category with the highest prevalence as reference, age as a continuous variable, and all other factors as dichotomous covariates. The statistical analysis software Stata 12.1, 2013 (StataCorp, College Station, TX, USA) was used for statistical calculations.

Whenever applicable, the REMARK recommendations for reporting of tumor marker studies were followed (McShane et al. 2006).

## Results

### Clinicopathological characteristics and outcome

The majority (57%) of the 192 patients had small (≤20 mm) tumours and axillary lymph node status (nodal status) was pathological node negative (pN-) in 106 (59%) (Table 1). Histological grade was 1 in 16%, 2 in 76% and 3 in 8%. One hundred and sixy nine patients (88%) were ER positive. The expression of Ki67 in the tumour was low in 115 patients (60%), intermediate in 62 (32%) and high in 15 (8%). Four patients (2%) were HER2-positive (Table 3). A total of 76 patients (40%) had a recurrence, and the distribution of site of first recurrence was: Local in 16 patients, regional in 6 and distant in 54. In addition 8% developed a contralateral BC (Table 2). At the end of the study, 60 patients (31%) had died from breast cancer and 72 (38%) from other causes. The remaining 60 patients (31%) were still alive and had a median follow-up of 21 years (range 0.7-30 years) (Table 2).

### Correlation between Ki67, histological grade and other prognostic factors

A significant positive correlation (*r* = 0.26) was seen between Ki67 and histological grade (*p* < 0.001), but not between Ki67 and other prognostic factors (nodal status, ER, PgR, age and tumour size). Histological grade was also significantly correlated to tumour size (*r* = 0.25, *p* = 0.001) but not to the other factors mentioned above.

### Breast cancer mortality

#### Univariable analyses

**Figure 3:**
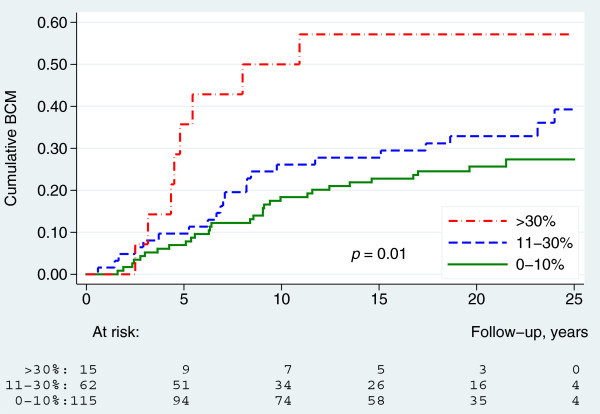
**Breast cancer mortality by Ki67 (** ***n*** **=192).**

**Figure 4:**
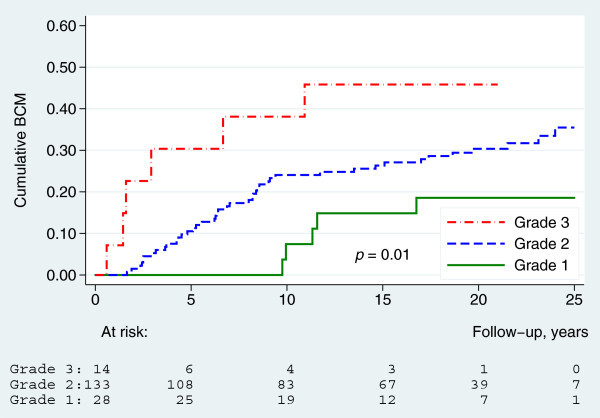
**Breast cancer mortality by histological grade (** ***n*** **=175).**

A log-rank test for trend showed significant differences in BCM between the three predefined Ki67 groups (*p* = 0.01). Furthermore, a Cox-regression analysis showed, that the BCM was significantly higher in the high Ki67-group compared to the low Ki67-group (hazard ratio (HR) = 2.6, 95% confidence interval (95% CI): 1.2-5.8, *p* = 0.01), whereas the difference between the intermediate Ki67-group and the low Ki67-group was non-significant (HR = 1.4, 95% CI: 0.84-2.5, *p* = 0.19) (Figure 3). Significant differences in BCM were also seen between the three different histological grades (*p* = 0.01). BCM was significantly higher in grade 3 compared to grade 2 (HR = 2.8, 95% CI: 1.2-6.7, *p* = 0.02) but not in grade 1 compared to grade 2 (HR = 0.54, 95% CI: 0.21-1.4, *p* = 0.19) (Figure 4).

When the three components of histological grade were analyzed separately by log-rank tests for trend, we found significant differences in BCM between the three different mitotic scores (*p* < 0.001), borderline significance between the three pleomorphism groups (*p* = 0.04) and non-significant differences for tubule formation (*p* = 0.70). In Cox-regression analyses, BCM was significantly higher in patients with larger tumours (>20 mm) compared to those with smaller (≤20 mm) (HR = 2.7, 95% CI: 1.6-4.5, *p* < 0.001), and also significantly higher in patients with more than 3 lymph node metastases compared to node negative patients (HR = 3.5, 95% CI: 2.0-6.5, *p* < 0.001), whereas the difference between patients with 1 to 3 lymph node metastases and node negative patients was non-significant (HR = 1.1, 95% CI: 0.55-2.4, *p* = 0.73). Age was also significantly associated with BCM, whereas ER and PgR, were not (Table 4).

**Table 4:** **Univariable analysis of breast cancer mortality in invasive lobular carcinoma**

Prognostic factor	n	# events	HR	95% CI	***p***-value
**Age** (years)	192	60	0.97	0.95-0.99	0.01
**Tumour size** (mm)					
≤20	108	24	1.0		
>20	81	36	2.7	1.6-4.5	<0.001
**Nodal status** (*p* <0.001)^a^					
0	107	27	1.0		
1-3+	36	10	1.1	0.55-2.4	0.73
>3+	38	21	3.5	2.0-6.2	<0.001
**Histological grade** (*p* =0.01)^a^					
1	28	5	0.54	0.21-1.4	0.19
2	133	43	1.0		
3	14	6	2.8	1.2-6.7	0.02
**Tubule formation**					
2	13	3	1.0		
3	162	51	1.3	0.39-4.0	0.70
**Pleomorphism** (*p* =0.04)^a^					
1	20	5	0.88	0.34-2.3	0.80
2	112	31	1.0		
3	43	18	1.9	1.0-3.3	0.03
**Mitotic score** (*p* <0.001)^a^					
1	155	45	1.0		
2	10	4	2.0	0.71-5.6	0.19
3	10	5	4.2	1.7-11	0.003
**ER**					
**+**	169	50	1.0		
**-**	23	10	1.6	0.83-3.3	0.15
**PgR**					
**+**	97	27	1.0		
**-**	95	33	1.4	0.85-2.4	0.18
**Ki67** (*p* = 0.01)^a^					
0-10%	115	30	1.0		
11-30%	62	22	1.4	0.84-2.5	0.19
>30%	15	8	2.6	1.2-5.8	0.01
**KiGE**					
Low	136	35	1.0		
High	39	19	2.8	1.6-5.0	<0.001

^a^Log-rank test for trend.

**Figure 5:**
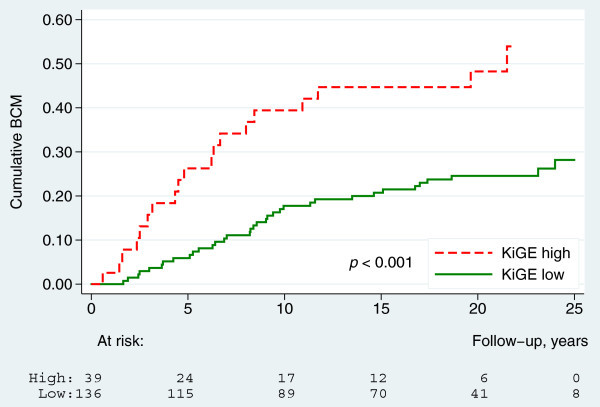
**Breast cancer mortality by KiGE (** ***n*** **=175).**

Based on our previous studies (Klintman et al. 2010; Strand et al. 2013), we hypothesized that a combination of Ki67, histological grade and ER provided stronger prognostic information than if these factors were used individually. We found also that in ILC, KiGE was a strong prognostic factor for BCM (Cox-regression, HR = 2.8, 95% CI: 1.6-5.0, *p* < 0.001) (Figure 5).

#### Multivariable analyses

In a multivariable model, including adjuvant treatment, age, tumour size, nodal status, histological grade, ER, PgR and Ki67, the only significant prognostic factor for BCM was nodal status (*p* < 0.001). More specifically, patients with more than 3 lymph node metastases had significantly higher BCM compared to node negative patients (HR = 4.0, 95% CI: 1.8-8.5, *p* <0.001). Even though histological grade was not significant when evaluated as a factor on three levels (*p* = 0.08), patients with histological grade 3 tumours had significantly higher BCM compared to patients with grade 2 tumours (HR = 3.2, 95% CI: 1.2-8.7, *p* = 0.02). All the other factors were non-significant (Table 5).

**Table 5:** **Multivariable analysis of breast cancer mortality in invasive lobular carcinoma** ^**a**^

Prognostic factor	n	# events	HR	95% CI	***p***-value
**Age** (years)	161	52	0.98	0.96-1.01	0.29
**Tumour size** (mm)					
≤20	93	21	1.0		
>20	68	31	1.8	0.91-3.7	0.09
**Nodal status** (*p* <0.001)^b^					
0	100	25	1.0		
1-3+	27	7	0.88	0.35-2.2	0.78
>3+	34	20	4.0	1.8-8.5	<0.001
**Histological grade** (*p* = 0.08)^b^					
1	25	5	1.1	0.41-3.0	0.83
2	124	41	1.0		
3	12	6	3.2	1.2-8.7	0.02
**ER**					
**+**	141	42	1.0		
**-**	20	10	1.1	0.52-2.4	0.77
**PgR**					
**+**	87	25	1.0		
**-**	74	27	1.3	0.76-2.4	0.31
**Ki67** (*p* = 0.36)^b^					
0-10%	92	25	1.0		
11-30%	57	21	1.3	0.72-2.5	0.36
>30%	12	6	2.0	0.73-5.5	0.18

^a^Adjusted for adjuvant treatment.

^b^2-degree-of-freedom test of no factor effect.

#### KiGE

If KiGE was used instead of Ki67, histological grade and ER, we found that KiGE (HR = 2.0, 95% CI: 1.1-3.6, *p* = 0.03) and nodal status (*p* = 0.003, 2-degree of freedom test) were independent long-term prognostic factors for BCM in multivariable analysis. Patients with >3 positive lymph nodes had significantly higher BCM than node negative patients (HR = 2.9, 95% CI: 1.4-6.1, *p* = 0.003). Tumour size (>20 mm vs. ≤20 mm) was also a prognostic factor, but not significant in this model (HR = 1.9, 95% CI: 0.98-3.8, *p* = 0.06) (Table 6).

**Table 6:** **Multivariable analysis of breast cancer mortality in invasive lobular carcinoma (including KiGE)** ^**a**^

Prognostic factor	n	# events	HR	95% CI	***p***-value
**Age** (years)	161	52	0.99	0.96-1.02	0.48
**Tumour size** (mm)					
≤20	93	21	1.0		
>20	68	31	1.9	0.98-3.8	0.06
**Nodal status** (*p* = 0.003)^b^					
0	100	25	1.0		
1-3+	27	7	0.87	0.35-2.2	0.77
>3**+**	34	20	2.9	1.4-6.1	0.003
**PgR**					
**+**	87	25	1.0		
**-**	74	27	1.4	0.78-2.4	0.27
**KiGE**					
Low	125	34	1.0		
High	36	18	2.0	1.1-3.6	0.03

^a^Adjusted for adjuvant treatment.

^b^2-degree-of-freedom test of no factor effect.

#### Combination index (KiGE-TN)

**Figure 6:**
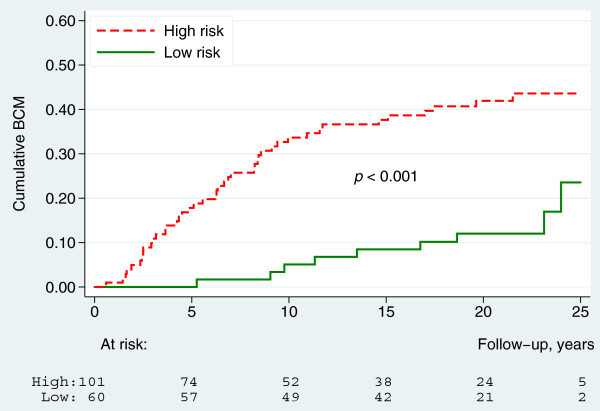
**Breast cancer mortality by KiGE-TN (** ***n*** **=161).**

The three strongest prognostic factors in the last multivariable analysis above were combined into a simplified classifier defined as presence of at least one of the three risk factors: Tumour size >20 mm, ≥1 positive lymph node and high-KiGE. Patients not displaying any of these risk factors had 10 and 20 years BCM of 5% (95% CI: 1-13%) and 12% (95% CI: 5-22%), respectively. This group consisted of 37% of the patients in the present study. The remaining 63% of the patients had 10 and 20 years BCM of 35% (95% CI: 26-44%) and 42% (95% CI: 32-51%), respectively (Figure 6).

## Discussion

This is a retrospective, population-based, long-term follow-up study, including patients with primary ILC, diagnosed and operated on between 1980 and 1991. The baseline patient- and tumour characteristics as well as clinical outcome data were comparable to those of earlier studies on ILC (Arpino et al. 2004; Pestalozzi et al. 2008; Orvieto et al. 2008; Rakha et al. 2008b). Surgery, radiotherapy and adjuvant systemic treatment at the time differ substantially from today’s guidelines. As a consequence mastectomy and ALND rates in this cohort are high (79% and 94% respectively) and only 44% of the ER and/or PgR positive patients received endocrine therapy (none of them received aromatase inhibitors), and despite the fact that 41% were node positive only 3% received chemotherapy. According to current treatment trends in breast cancer, the patients in our cohort had “local over-treatment” and a “systemic under-treatment”. Possible consequences are a higher degree of surgery related sequelae and higher frequency of both early and late distant recurrences. Concurrently, we saw a low frequency of local recurrences (11%) and the fact that so few of the patients received endocrine and/or chemotherapy gives us an exclusive opportunity to study untreated ILC, close to the natural history of ILC, in a way not possible today. Distant recurrences, resulting in BC death, are common in this cohort. Many of the recurrences occur during 10–20 years of FU and some of them over 20 years past diagnosis, which is also seen in other studies (Pestalozzi et al. 2008; Rakha et al. 2008b), mirroring the chronic history of the disease and that early detection is not directly linked to good long-term prognosis for the individual patient.

The main purpose of this study was to evaluate the long-term impact of different prognostic factors in ILC, with a primary focus on Ki67 and histological grade alone, and in combination with, ER. Ki67 was a significant prognostic factor in univariable analysis but did not reach significance after adjustment for adjuvant treatment and other known prognostic factors. In an earlier ILC-study with a shorter follow-up time (7 years), no significant prognostic effect of Ki67 on overall survival could be detected (Orvieto et al. 2008) and to our knowledge there is, to date, no other comparable study, investigating the long-term prognostic effect of Ki67 in ILC. Studies conducted on Ki67 in mixed BC materials have shown an independent prognostic significance (Urruticoechea et al. 2005; de Azambuja et al. 2007). The combination of Ki67, histological grade and ER into KiGE however, gives useful independent long-term prognostic information in ILC. High-KiGE ILC’s (20%) had a 10 and 20 years BCM of 40% and 48%, respectively and the remaining low-KiGE ILC’s (80%) had a 10 and 20 years BCM of 19% and 25%, respectively. Patients with high-KiGE ILC’s in this cohort represent an under-treated group, with poor prognostic tumours, and today the majority would have been recommended adjuvant chemotherapy to improve outcome. Patients with low-KiGE ILC’s, on the other hand, represent the greater part of this cohort, containg tumours with a more diversified prognosis, causing difficulties in adjuvant treatment decision-making. Based on information from our multivariable analyses, which showed that both KiGE, tumour size and nodal status were strong prognostic factors, we divided the low-KiGE ILC’s further, resulting in an extremely low-risk group (low-KiGE, T1, N0) consisting of 37% of the patients with a long-term prognosis of BCM 5% at 10 years and 12% at 20 years follow-up. None of these patients recieved chemotherapy and only 2 recieved endocrine treatment with tamoxifen. These findings suggest that this group could be spared from both adjuvant chemo- and endocrine therapy, even though there might be a possibility to improve the long-term outcome even further by giving endocrine therapy in accordance with modern guidelines. Nodal status was the strongest independent prognostic factor in this material, which is in concert with previous observations in ILC materials (Arpino et al. 2004; Wasif et al. 2010). Surprisingly, there was no significant difference in outcome between node negative patients and those with 1–3 lymph node metastasis, and a plausible explanation is that the node negative group in this cohort, harbours a portion of false negative patients due to the more inexact pathology examination methods used at the time of diagnosis, compared to today’s. ER status was of limited prognostic value, probably mainly because so few of the patients were ER negative (12%). Histological grading in BC is built around three components: Tubule formation, pleomorphism and mitotic rate. Normally tubule formation is absent in most ILC’s, which means there are only two parameters left to rely on when dividing ILC’s into different grades, and pathologists have historically been reluctant to grade ILC. In spite of this, and also the fact that the majority of the ILC’s in this study were classified as grade 2 (76%), histological grade was an independent long-term prognostic factor. The histological grade component with greatest significance was mitotic score, bearing much of the prognostic information which was also consistent over time. Pleomorphism seems to bear significant prognostic information particularly in short-term, but the effect weakens considerably over time. As proposed tubule formations were absent in most of the tumours (93%) and this component added limited prognostic information. These results are consistent with those found in a previous long-term follow-up study focusing on histological grade and its components in ILC (Rakha et al. 2008b). A finding from our long-term data worth noting is, that the ILC’s in the extremely low-risk group (low-KiGE, pT0, pN0) have a slightly higher BCM year 10–20 (7%), compared to the first 10 years (5%) of follow-up indicating that, even in this group of patients with a very good overall prognosis, improvements in the adjuvant management is still needed in order to prevent late distant recurrences and breast cancer death.

The ATLAS-study, comparing 10 vs. 5 years of adjuvant tamoxifen in a mixed BC material, shows an advantage for longer duration of treatment in reducing late recurrences with a long-term benefit mainly after 10 years (Davies et al. 2012). Furthermore, recent data from BIG 1–98 study, presented at the San Antonio Breast Cancer Symposium 2012, comparing tamoxifen vs. letrozole as adjuvant therapy in primary ILC, show a significant benefit from letrozol treatment regardless of proliferation status, but with an even greater effect in high-proliferative ILC, suggesing letrozole as a potential up-front adjuvant treatment in all ILC’s. Prolonged endocrine therapy and up-front treatment with aromatase inhibitors, in order to prevent late recurrences and improve long-term outcome in ILC, is an interesting issue and still an open question. Ki67 cut-offs in this study were chosen based on the knowledge from repetitive studies focusing on the reproductibility of Ki67 analysis and scoring, showing that there is still a disconcordance between different laboratories, particularly in the intermediate interval (11-30%) (Dowsett et al. 2011; Gudlaugsson et al. 2012). ILC’s with Ki67 >30% were classified as high-proliferative. With these cut-off values we hoped to identify the “true” high-proliferative tumours even though important prognostic information from the intermediate group (Ki67 11-30%) might have been lost. Taking a closer look at this retrospective ILC-material, the division of the tumours into low- and high-KiGE is, to some extent, similar to the more modern division of BC’s into luminal subtypes according to the 2013 St Gallen guidelines, since many of the ILC would be included in the luminal A and luminal B groups (Goldhirsch et al. 2013).

In conclusion our results show that the well-established prognostic factors in BC, including Ki67, are still valid for ILC’s, and the combination of Ki67, histological grade and ER into KiGE, together with tumour size and nodal status makes it possible to identify a large group of ILC patients (37%) with a long-term natural history associated with such a good prognosis, that chemotherapy can be safely avoided and exclusion of endocrine therapy considered.

### Ethical standards

The study was approved by the ethics committee at Lund University (LU 240–01).

## References

[CR1] Acs G, Lawton TJ, Rebbeck TR, LiVolsi VA, Zhang PJ (2001). Differential expression of E-cadherin in lobular and ductal neoplasms of the breast and its biologic and diagnostic implications. Am J Clin Pathol.

[CR2] Arpino G, Bardou VJ, Clark GM, Elledge RM (2004). Infiltrating lobular carcinoma of the breast: tumor characteristics and clinical outcome. Breast Cancer Res.

[CR3] Barnes DM, Lammie GA, Millis RR, Gullick WL, Allen DS, Altman DG (1988). An immunohistochemical evaluation of c-erbB-2 expression in human breast carcinoma. Br J Canc.

[CR4] Biglia N, Mariani L, Sgro L, Mininanni P, Moggio G, Sismondi P (2007). Increased incidence of lobular breast cancer in women treated with hormone replacement therapy: implications for diagnosis, surgical and medical treatment. Endocr Relat Cancer.

[CR5] Davies C, Pan H, Godwin J, Gray R, Arriagada R, Raina V, Abraham M, Alencar VH, Badran A, Bonfill X, Bradbury J, Clarke M, Collins R, Davis SR, Delmestri A, Forbes JF, Haddad P, Hou MF, Inbar M, Khaled H, Kielanowska J, Kwan WH, Mathew BS, Mittra I, Muller B, Nicolucci A, Peralta O, Pernas F, Petruzelka L, Pienkowski T, Radhika R, Rajan B, Rubach MT, Tort S, Urrutia G, Valentini M, Wang Y, Peto R (2012). Long-term effects of continuing adjuvant tamoxifen to 10 years versus stopping at 5 years after diagnosis of oestrogen receptor-positive breast cancer: ATLAS, a randomised trial. Lancet.

[CR6] de Azambuja E, Cardoso F, de Castro G, Colozza M, Mano MS, Durbecq V, Sotiriou C, Larsimont D, Piccart-Gebhart MJ, Paesmans M (2007). Ki-67 as prognostic marker in early breast cancer: a meta-analysis of published studies involving 12,155 patients. Br J Cancer.

[CR7] Dowsett M, Nielsen TO, A’Hern R, Bartlett J, Coombes RC, Cuzick J, Ellis M, Henry NL, Hugh JC, Lively T, McShane L, Paik S, Penault-Llorca F, Prudkin L, Regan M, Salter J, Sotiriou C, Smith IE, Viale G, Zujewski JA, Hayes DF (2011). Assessment of Ki67 in breast cancer: recommendations from the International Ki67 in Breast Cancer working group. J Natl Cancer Inst.

[CR8] Elston CW, Ellis IO (1991). Pathological prognostic factors in breast cancer. I. The value of histological grade in breast cancer: experience from a large study with long-term follow-up. Histopathology.

[CR9] Frost AR, Terahata S, Yeh IT, Siegel RS, Overmoyer B, Silverberg SG (1995). An analysis of prognostic features in infiltrating lobular carcinoma of the breast. Mod Pathol.

[CR10] Goldhirsch A, Ingle JN, Gelber RD, Coates AS, Thurlimann B, Senn HJ (2009). Thresholds for therapies: highlights of the St Gallen International Expert Consensus on the primary therapy of early breast cancer 2009. Ann Oncol.

[CR11] Goldhirsch A, Winer EP, Coates AS, Gelber RD, Piccart-Gebhart M, Thurlimann B, Senn HJ (2013). Personalizing the treatment of women with early breast cancer: highlights of the St Gallen International Expert Consensus on the Primary Therapy of Early Breast Cancer 2013. Ann Oncol.

[CR12] Gudlaugsson E, Skaland I, Janssen EA, Smaaland R, Shao Z, Malpica A, Voorhorst F, Baak JP (2012). Comparison of the effect of different techniques for measurement of Ki67 proliferation on reproducibility and prognosis prediction accuracy in breast cancer. Histopathology.

[CR13] Klintman M, Bendahl PO, Grabau D, Lovgren K, Malmstrom P, Ferno M (2010). The prognostic value of Ki67 is dependent on estrogen receptor status and histological grade in premenopausal patients with node-negative breast cancer. Mod Pathol.

[CR14] Lakhani SR, Ellis IO, Schnitt SJ, Tan PH, Vijver van de MJ (2012). WHO Classification of Tumours of the Breast.

[CR15] Li CI, Anderson BO, Daling JR, Moe RE (2003). Trends in incidence rates of invasive lobular and ductal breast carcinoma. JAMA.

[CR16] Martinez V, Azzopardi JG (1979). Invasive lobular carcinoma of the breast: incidence and variants. Histopathology.

[CR17] McShane LM, Altman DG, Sauerbrei W, Taube SE, Gion M, Clark GM (2006). REporting recommendations for tumor MARKer prognostic studies (REMARK). Breast Cancer Res Treat.

[CR18] Moreno-Elola A, Aguilar A, Roman JM, Hernandez A, Martin M, Diaz Rubio E, Furio V, Fernandez C, De La Fuente P, San Roman JM, Escudero M (1999). Prognostic factors in invasive lobular carcinoma of the breast: a multivariate analysis. A multicentre study after seventeen years of follow-up. Ann Chir Gynaecol.

[CR19] Olsson S, Andersson I, Karlberg I, Bjurstam N, Frodis E, Hakansson S (2000). Implementation of service screening with mammography in Sweden: from pilot study to nationwide programme. J Med Screen.

[CR20] Orvieto E, Maiorano E, Bottiglieri L, Maisonneuve P, Rotmensz N, Galimberti V, Luini A, Brenelli F, Gatti G, Viale G (2008). Clinicopathologic characteristics of invasive lobular carcinoma of the breast: results of an analysis of 530 cases from a single institution. Cancer.

[CR21] Pestalozzi BC, Zahrieh D, Mallon E, Gusterson BA, Price KN, Gelber RD, Holmberg SB, Lindtner J, Snyder R, Thurlimann B, Murray E, Viale G, Castiglione-Gertsch M, Coates AS, Goldhirsch A (2008). Distinct clinical and prognostic features of infiltrating lobular carcinoma of the breast: combined results of 15 International Breast Cancer Study Group clinical trials. J Clin Oncol.

[CR22] Petrelli F, Barni S (2013). Response to neoadjuvant chemotherapy in ductal compared to lobular carcinoma of the breast: a meta-analysis of published trials including 1,764 lobular breast cancer. Breast Canc Res Treat.

[CR23] Rakha EA, El-Sayed ME, Powe DG, Green AR, Habashy H, Grainge MJ, Robertson JF, Blamey R, Gee J, Nicholson RI, Lee AH, Ellis IO (2008). Invasive lobular carcinoma of the breast: response to hormonal therapy and outcomes. Eur J Cancer.

[CR24] Rakha EA, El-Sayed ME, Menon S, Green AR, Lee AH, Ellis IO (2008). Histologic grading is an independent prognostic factor in invasive lobular carcinoma of the breast. Breast Cancer Res Treat.

[CR25] Rakha EA, Patel A, Powe DG, Benhasouna A, Green AR, Lambros MB, Reis-Filho JS, Ellis IO (2010). Clinical and biological significance of E-cadherin protein expression in invasive lobular carcinoma of the breast. Am J Surg Pathol.

[CR26] Reeves GK, Beral V, Green J, Gathani T, Bull D (2006). Hormonal therapy for menopause and breast-cancer risk by histological type: a cohort study and meta-analysis. Lancet Oncol.

[CR27] Sastre-Garau X, Jouve M, Asselain B, Vincent-Salomon A, Beuzeboc P, Dorval T, Durand JC, Fourquet A, Pouillart P (1996). Infiltrating lobular carcinoma of the breast. Clinicopathologic analysis of 975 cases with reference to data on conservative therapy and metastatic patterns. Cancer.

[CR28] Shi SR, Key ME, Kalra KL (1991). Antigen retrieval in formalin-fixed, paraffin-embedded tissues: an enhancement method for immunohistochemical staining based on microwave oven heating of tissue Sections. J Histochem Cytochem.

[CR29] Singletary SE, Patel-Parekh L, Bland KI (2005). Treatment trends in early-stage invasive lobular carcinoma: a report from the National Cancer Data Base. Ann Surg.

[CR30] Sinha PS, Bendall S, Bates T (2000). Does routine grading of invasive lobular cancer of the breast have the same prognostic significance as for ductal cancers?. Eur J Surg Oncol.

[CR31] Strand C, Ahlin C, Bendahl PO, Fjallskog ML, Hedenfalk I, Malmstrom P, Ferno M (2011). Combination of the proliferation marker cyclin A, histological grade, and estrogen receptor status in a new variable with high prognostic impact in breast cancer. Breast Cancer Res Treat.

[CR32] Strand C, Bak M, Borgquist S, Chebil G, Falck AK, Fjallskog ML, Grabau D, Hedenfalk I, Jirstrom K, Klintman M, Malmstrom P, Olsson H, Ryden L, Stal O, Bendahl PO, Ferno M (2013). The combination of Ki67, histological grade and estrogen receptor status identifies a low-risk group among 1,854 chemo-naive women with N0/N1 primary breast cancer. SpringerPlus.

[CR33] Talman ML, Jensen MB, Rank F (2007). Invasive lobular breast cancer. Prognostic significance of histological malignancy grading. Acta Oncol.

[CR34] Urruticoechea A, Smith IE, Dowsett M (2005). Proliferation marker Ki-67 in early breast cancer. J Clin Oncol.

[CR35] Wachtel MS, Halldorsson A, Dissanaike S (2010). Nottingham grades of lobular carcinoma lack the prognostic implications they bear for ductal carcinoma. J Surg Res.

[CR36] Wasif N, Maggard MA, Ko CY, Giuliano AE (2010). Invasive lobular vs. ductal breast cancer: a stage-matched comparison of outcomes. Ann Surg Oncol.

[CR37] Wiesner FG, Magener A, Fasching PA, Wesse J, Bani MR, Rauh C, Jud S, Schrauder M, Loehberg CR, Beckmann MW, Hartmann A, Lux MP (2009). Ki-67 as a prognostic molecular marker in routine clinical use in breast cancer patients. Breast.

